# Burying Alive

**Published:** 1881-01

**Authors:** 


### BURYING ALIVE.

IN a country village in the central part of this State, the people were considerably agitated a few months ago on the question as to whether a young woman, who had remained motionless and apparently breathless for several days, was really dead or only in a condition of trance. It seems that she seemed to die suddenly of heart disease ; but when tbe body was being prepared for burial it was found that the cheeks were flushed, and the flesh seemed natural and pliable, as if she were only asleep, and not cold and stiff as in death. The case excited great interest throughout the country, and people were anxious to know what the result would be. After several days of suspense it was announced that the question had been settled by burying the poor girl. This was all the information to be had in relation to a case in which thousands of people had taken a great interest.

Now a similar case is reported from Baltimore, January 5th : "To-day the friends of Miss Barbara Liefield, the daughter of a wealthy German, who died on Friday last, placed a watch over her coffin, believing that she is in a trance and will come to life again. Miss Liefield, who was about eighteen years of age, was found dead in bed, and her physician said she died of heart disease. On Sunday she was to have been buried, but, to the astonishment of her friends, her face assumed a life-like hue, and the undertaker expressed the opinion that the body had turned partly on its side. Consequently the funeral was indefinitely postponed, and further developments were awaited with intense interest. The cheeks assumed a rosy hue and the limbs were neither cold nor stiff. The hands were warm, and up to yesterday morning a warm perspiration was perceptible. Yesterday the body rapidly became cold, and several physicians who examined it pronounced life extinct. The funeral, accordingly, took place in the afternoon, the coffin being placed in a vault in St. Alphonsus Cemetery. To-day friends visited the vault and had the coffin opened. They were greatly agitated when they learned that the body had again become warm and that the color had returned to the cheeks. By the advice of the cemetery authorities the remains were not removed. The coffin was, however, left open, and a guard placed in the vault. The painful suspense under which the parents of the girl are laboring has completely prostrated them. The result is looked for with much anxiety."

One morning, several years ago, I called on a young physician who had been practicing a short time in a city in the western part of the state. While talking with him, a girl, of perhaps twelve years, came into his office, and, with tears streaming from her eyes, implored him to go and make a careful examination of her mother; for, said she, I believe her to be alive; there is color in her cheeks, and her limbs are not cold and stiff, but she seems as if she were only asleep. I can never forget the conduct of this young doctor as he arose from his chair, and, with harsh words, fairly drove the trembling child from his office. I attempted to leave at the same instant, but he, perhaps, suspecting my motive, detained me for a moment by asking some frivolous question, and when I got to the street the child was nowhere to be seen. I knew of no way to find out where the family lived, or who they were, for I knew the doctor would not tell me. The doctor had pronounced the woman dead; the funeral had been appointed; and, if it had turned out that he was mistaken, it might have been very mortifying to his vanity.

There is a natural fear on the part of every human being of being buried alive. The question is seldom discussed in the family and among friends without mutual requests that in case of death they may not be buried too soon.

When Washington realized that death was near, he requested that he might not be buried under two days from the time of death. Decomposition is the only infallible sign that life is extinct.

Without that—except in cases of injuries that destroy some vital organ—no one can say positively that the spirit has flown. It is probable that premature burials are very rare, except during some terrible epidemic. It is not pleasant, however, to think they are possible at any time.

When a member of the family dies, the relatives are often so stricken down with their sorrow that they are incapable of directing affairs. Too much discretion is given to the undertaker. It would generally be better to delay a little before sending for him and his ice-box. I have often met a gentleman named Bell, who, during the season of cholera in 1832, made his escape from a coffin while being conveyed to the cemetery. He said that he never lost consciousness while they were placing him in the coffin, but he had not the power to move sufficiently to show that he was alive; and so great was the panic that patients were not carefully examined to see if they were really dead, and the disease was so generally fatal that the undertakers seemed to think if their patients were not dead when they started for the grave-yard they surely would be by the time they got there. Mr. Bell lived many years afterward, and, indeed, may be living still. We occasionally hear that a grave has been opened and that the occupant has been found lying on the side or face. There is nothing astonishing about that. It may easily occur by the coffin being tilted as it is lowered into the grave ; but the most astonishing thing is the explanation that it is often sought to be given : that the muscles had been contracted by galvanic action and the body turned in that way. If a battery is applied that is possible ; but who can believe in spontaneous galvanic action on muscles that are rigid in death.

But to set all doubts and fears on this subject at rest, it would be well to prohibit by law all burials, until competent physicians had examined the bodies and found unmistakable signs of decomposition. With the modern appliances of ice and embalming fluids, bodies may be kept for days after decay has commenced, and thus all doubts and fears of loving friends set for ever at rest.

---

Furniture Polish.—For a polish to clean up and brighten old furniture, pianos, etc., dissolve four ounces of orange shellac in one quart of ninety-five per cent. alcohol ; to this add one quart of linseed oil and one pint of turpentine ; when mixed add four ounces of sulphuric ether and four ounces of aqua ammonia ; mix thoroughly before using. Apply with a cloth or sponge, and rub the surface to which it is applied until the polish appears.

